# Supplementary material for: Structural basis for the biosynthesis of lovastatin
Source: Nat Commun. 2021 Feb 8;12:867. doi: 10.1038/s41467-021-21174-8 (PMC7870829; doi:10.1038/s41467-021-21174-8)
Supplement: Supplementary file 1 — Supplementary Information [file 41467_2021_21174_MOESM1_ESM.pdf]

Supplementary Information

Structural basis for the biosynthesis of Lovastatin

Jialiang Wang[1], Jingdan Liang[1], Lu Chen[1], Wei Zhang[1], Liangliang Kong[2], Chao Peng[2], Chen Su[2], Yi Tang[3], Zixin Deng[1,*] & Zhijun Wang[1,*]

[1]State Key Laboratory of Microbial Metabolism and School of Life Science & Biotechnology, Shanghai Jiao Tong University, Shanghai, China.

[2]National Facility for Protein Science in Shanghai, Shanghai, China.

[3]Department of Chemical and Biomolecular Engineering and Department of Chemistry and Biochemistry, University of California, Los Angeles, California 90095, United States.

*Address correspondence to Zixin Deng, zxdeng@sjtu.edu.cn or Zhijun Wang, wangzhijun@sjtu.edu.cn

J.L.Wang and J.D.Liang contributed equally to this work.

This file includes:

Supplementary Fig. 1-13

Supplementary Table 1-3

Supplementary Reference

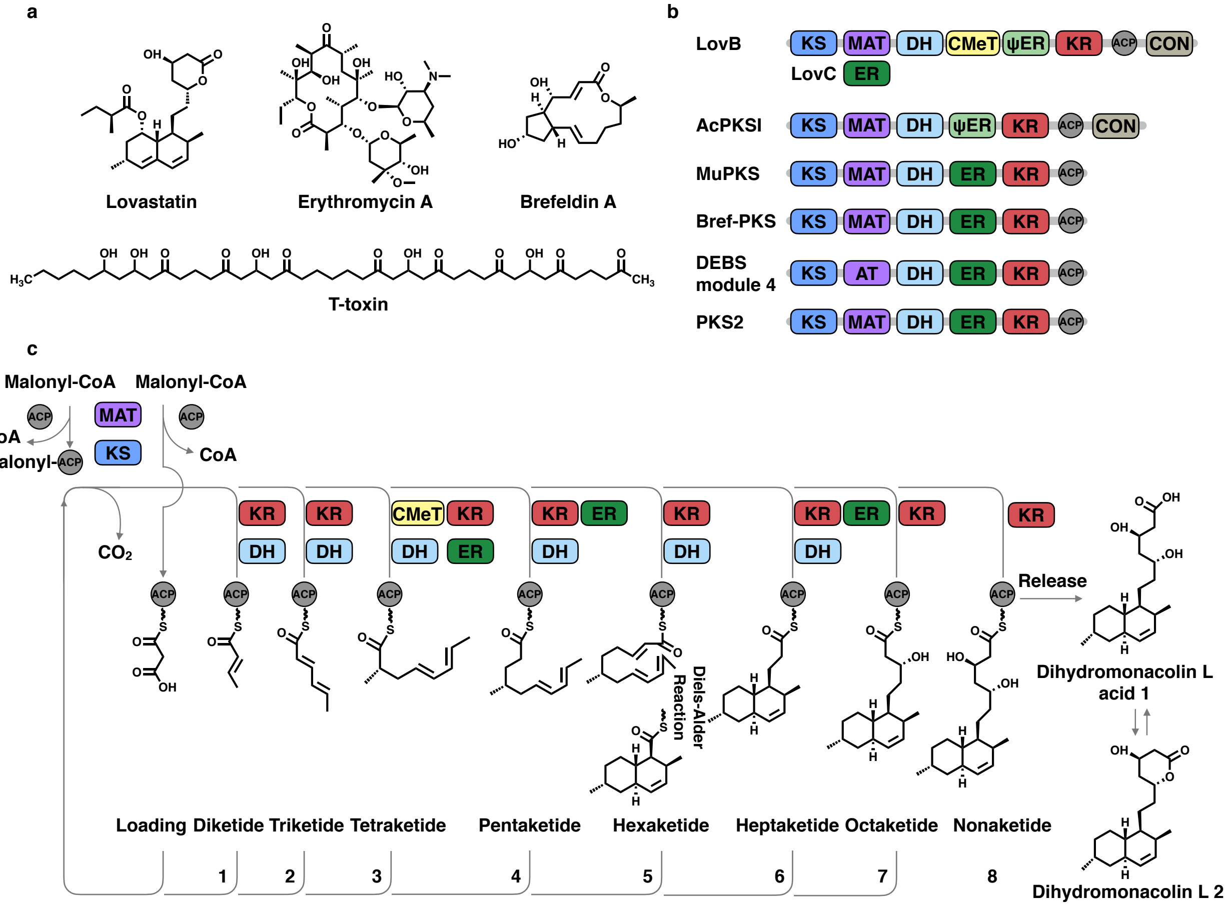

Supplementary Fig. 1 Representative PKs, PKSs, and the biosynthetic pathway of DML. a Polyketide examples. **b** Domain organizations for PKSs. LovB, lovastatin nonaketide synthase; LovC, *trans*-acting enoyl reductase; AcPKSI, PKS from *Aplysia californica*. MuPKS, PKS from Budgerigar. Bref-PKS, brefeldin polyketide synthase (PKS); DEBS module 4, 6-deoxyerythronolide B synthase module4; PKS2, PKS that mediates T-toxin biosynthesis. KS, β-ketoacyl synthase; AT, acyltransferase; MAT, malonyltransferase; DH, dehydratase; CMeT, methyltransferase; ER, enoyl reductase; KR, ketoreductase; ACP, acyl carrier protein; TE, thioesterase; and CON, condensation domain from nonribosomal peptide synthetases. Inactive pseudodomains are denoted by "ψ". **c** The proposed biosynthetic cycles by which LovB and LovC generate DML. The cycle is initiated by loading a malonyl-CoA, presumably followed by decarboxylation to generate the starter acetyl unit. Chain elongation is performed iteratively by eight rounds of Claisen condensation catalyzed by the KS domain and one Diels-Alder reaction. After each condensation reaction, the growing polyketide intermediate tethered to the phosphopantetheinyl arm of the ACP domain (shown as wavy lines) is subjected to reductive processing, which involves different permutations of CMeT-KR-DH-ER (LovC) usage. After the nonaketide is formed, the chain is released to generate dihydromonacolin L acid 1, which can then undergo lactonization to generate dihydromonacolin L 2. The cycles and the Diels-Alder reaction are indicated by numbers 1-8.

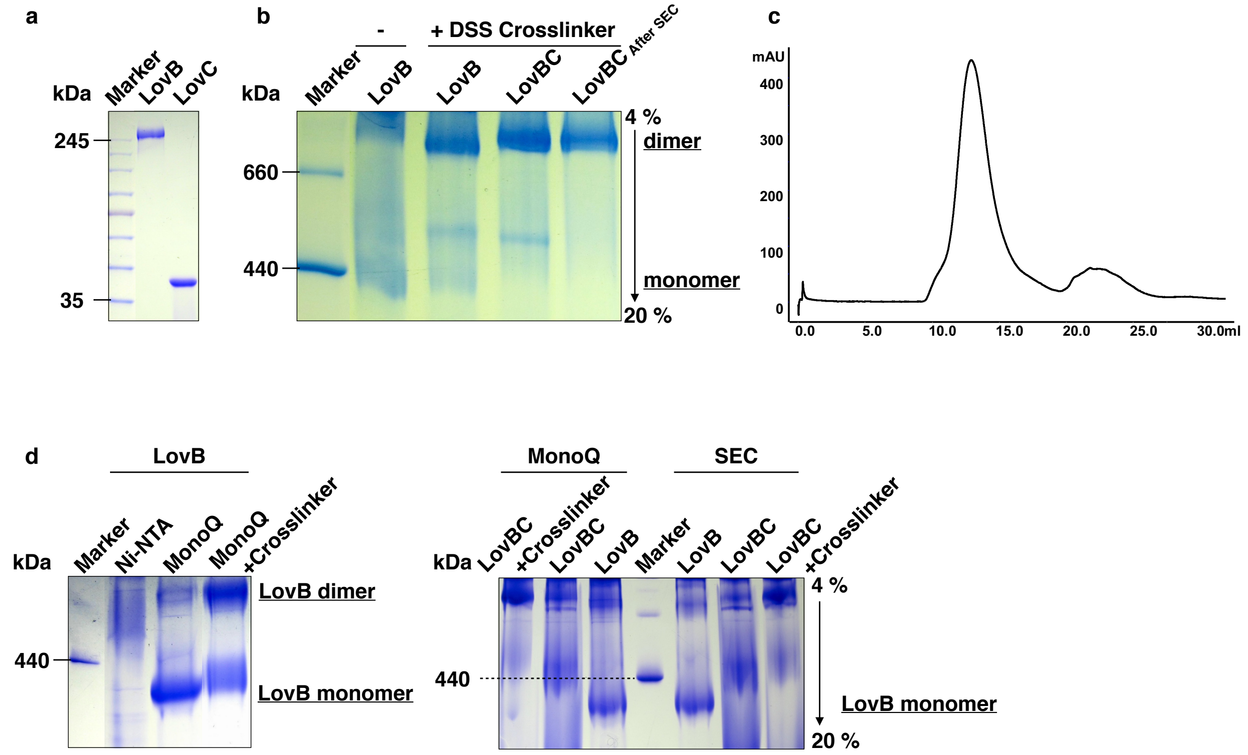

Supplementary Fig. 2 Purification of the LovBC complex. a SDS-PAGE analysis of Ni-NTA purified LovB and LovC. **b** Native-PAGE analysis of LovB and LovBC. A 4-20% gradient was used. LovB or LovBC was treated with a disuccinimidyl suberate (DSS) crosslinker before loading on the gel. **c** Size exclusion chromatography (SEC, Superose 6 Increase) profile of the crosslinked LovBC complex used for cryo-EM**. d** Native-PAGE analysis of LovB and LovBC after an ion exchange or a size exclusion chromatography purification step. Note that after the MonoQ step, there are more monomeric LovB contaminants. Therefore, this purification step was not employed in the current protocol. One representative result from at least three independent experiments is shown (a, b, d). Source data are provided as a Source Data file.

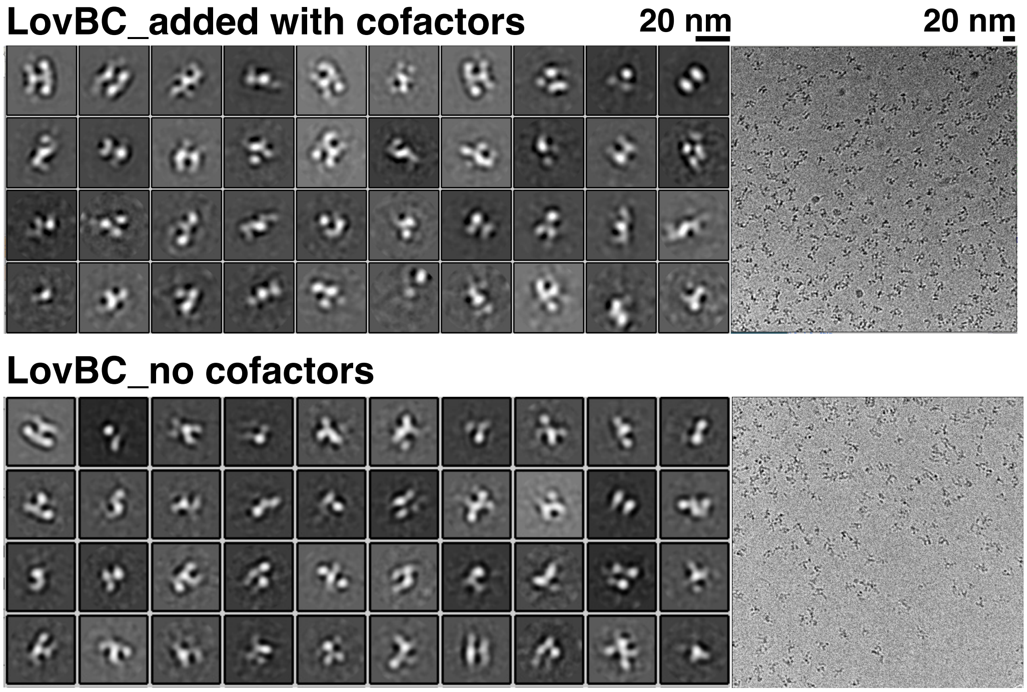

Supplementary Fig. 3 LovBC cryo-EM sample optimization. Two representative micrographs from independently collected forty micrographs and 2D classifications of protein particles prepared in the presence or absence of the cofactors (2 mM NADPH, 2 mM SAM, and 2 mM Malonyl-CoA). Scale bar, 20 nm.

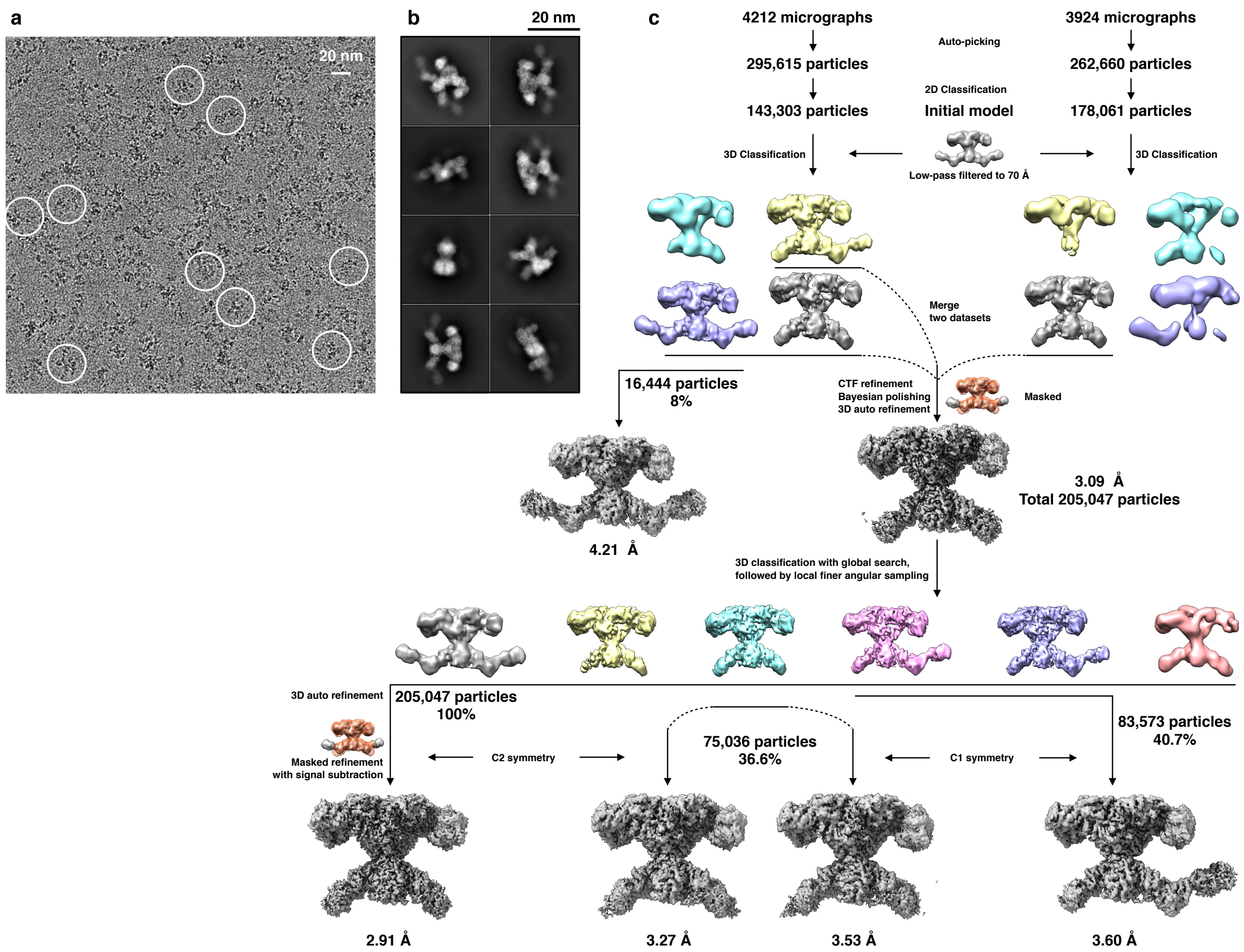

Supplementary Fig. 4 Cryo-EM structure determination of the LovBC complex. a One representative cryo-EM micrograph from the 8136 movie stacks of the LovBC complex with selected particles in white circles. Scale bar, 20 nm. **b** Eight representative 2D class averages of the LovBC complex from 100 averages. Scale bar, 20 nm. **c** A data processing workflow for the resolution-labeled density maps.

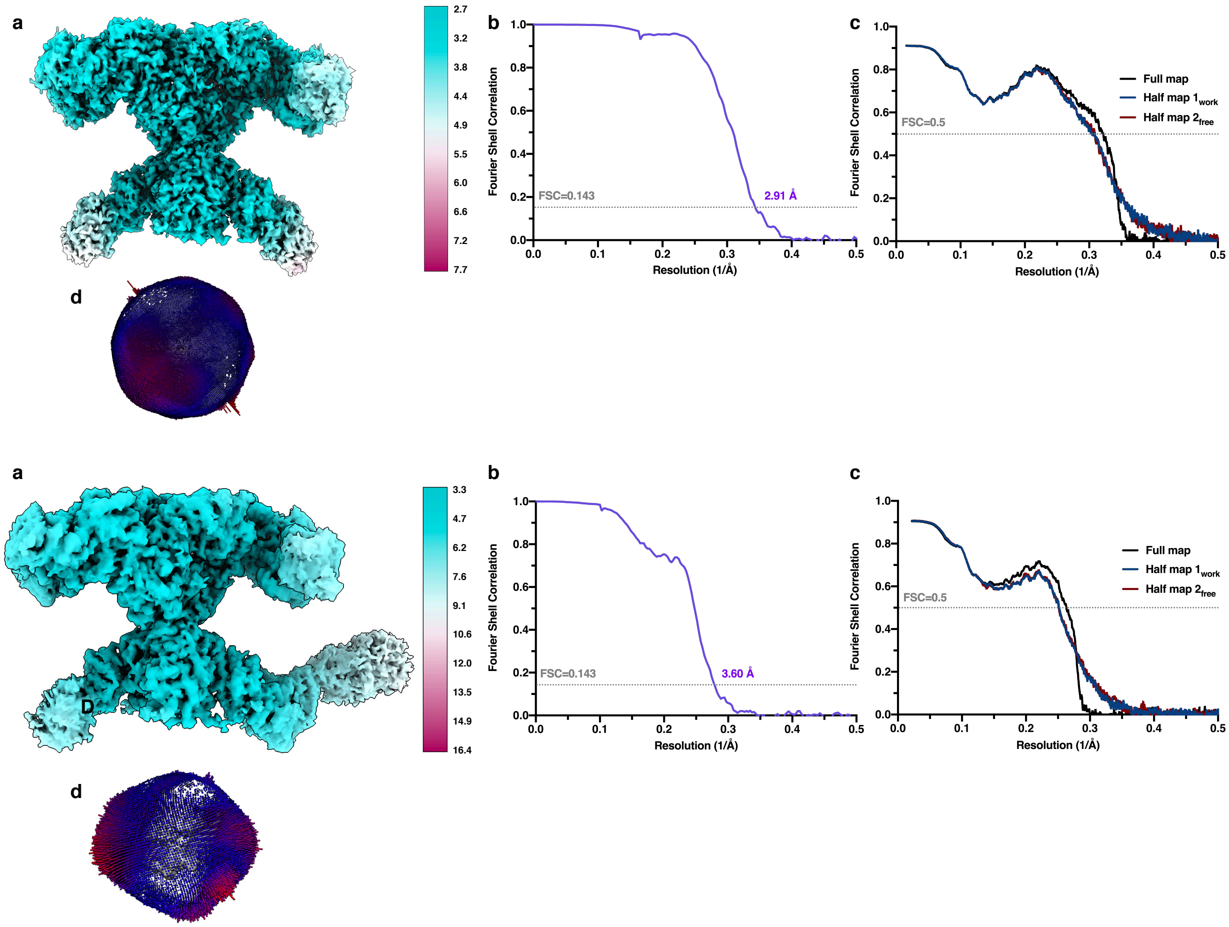

Supplementary Fig. 5 Cryo-EM analysis of the LovBC complex. Top, LovB; bottom, LovBC complex (with one side of LovC). **a** Local resolution of each map estimated in RELION. **b** Gold-standard FSC curves of the resolution-labeled density map (FSC=0.143 criterion). **c** FSC curves of the final refined model versus the map that it was refined against (black); of the model refined in the first of the two independent maps used for the gold-standard FSC versus that same map (dark blue); and of the model refined in the first of the two independent maps versus the second independent map (dark red). The small difference between the work and free FSC curves indicates that the model did not suffer from overfitting. **d** Angular distribution of all particles used for the final reconstruction of each map.

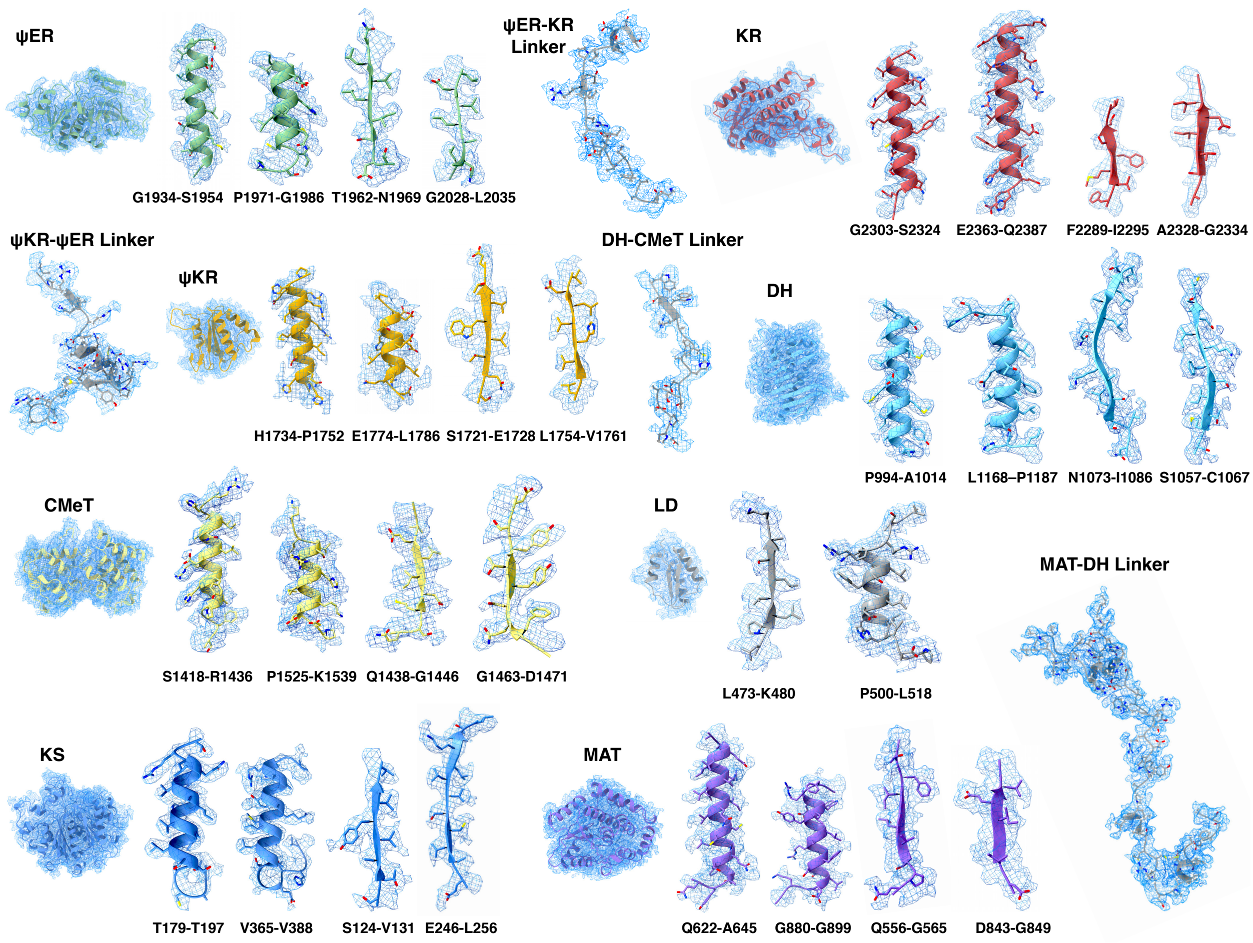

Supplementary Fig. 6 Cryo-EM density map of LovB. Close-up view of the density map for all the components of LovB (labeled, shown in blue transparent mesh), fitted with the atomic model. Representative small regions and linkers are shown in sticks with side chains.

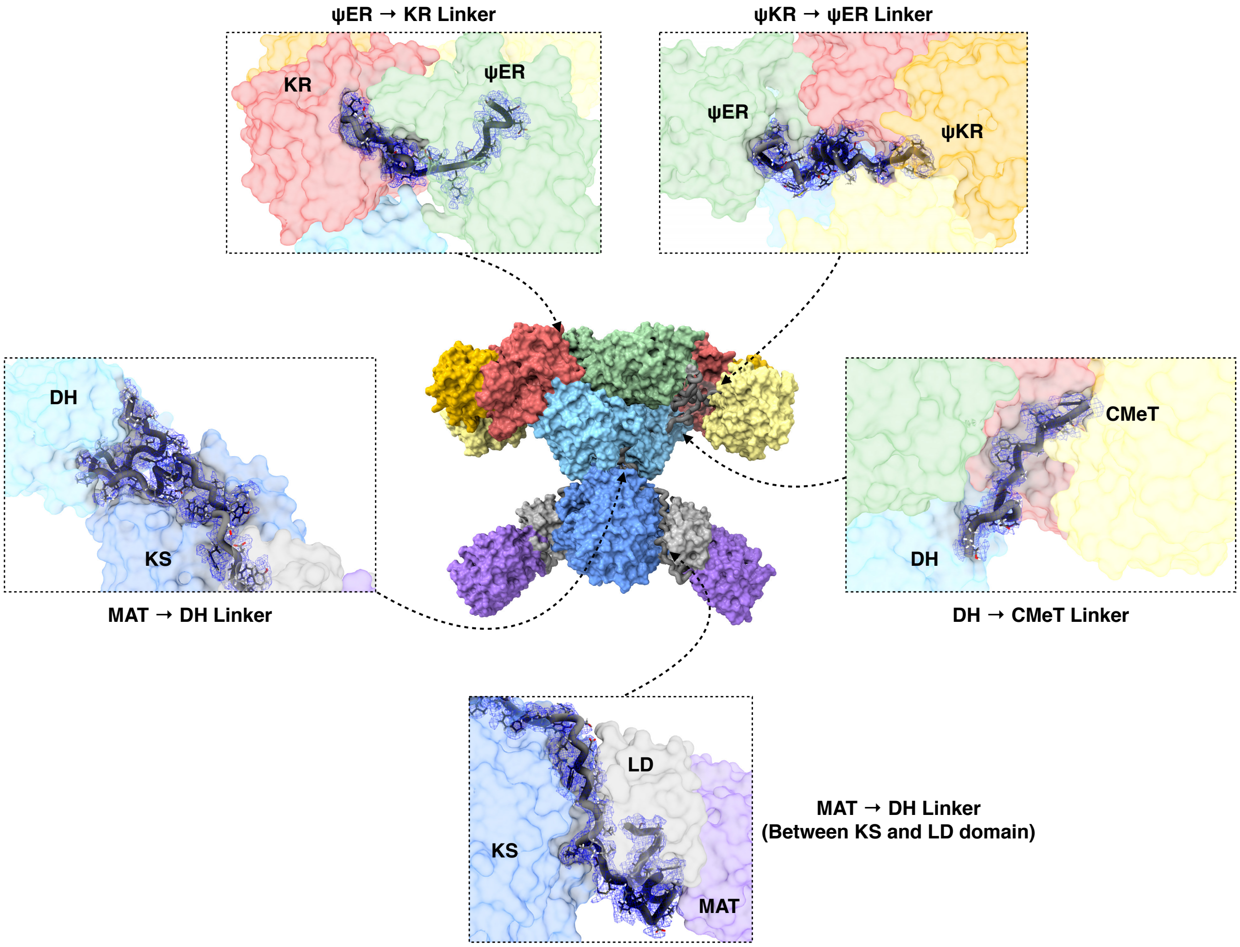

Supplementary Fig. 7 Linker-based domain organization of LovB. Surface representation of LovB, with domains colored as in Fig. 1a. Interdomain linkers are highlighted as thick gray oval tubes. The linker EM densities with corresponding atomic coordinates (as sticks) are shown in close-up view.

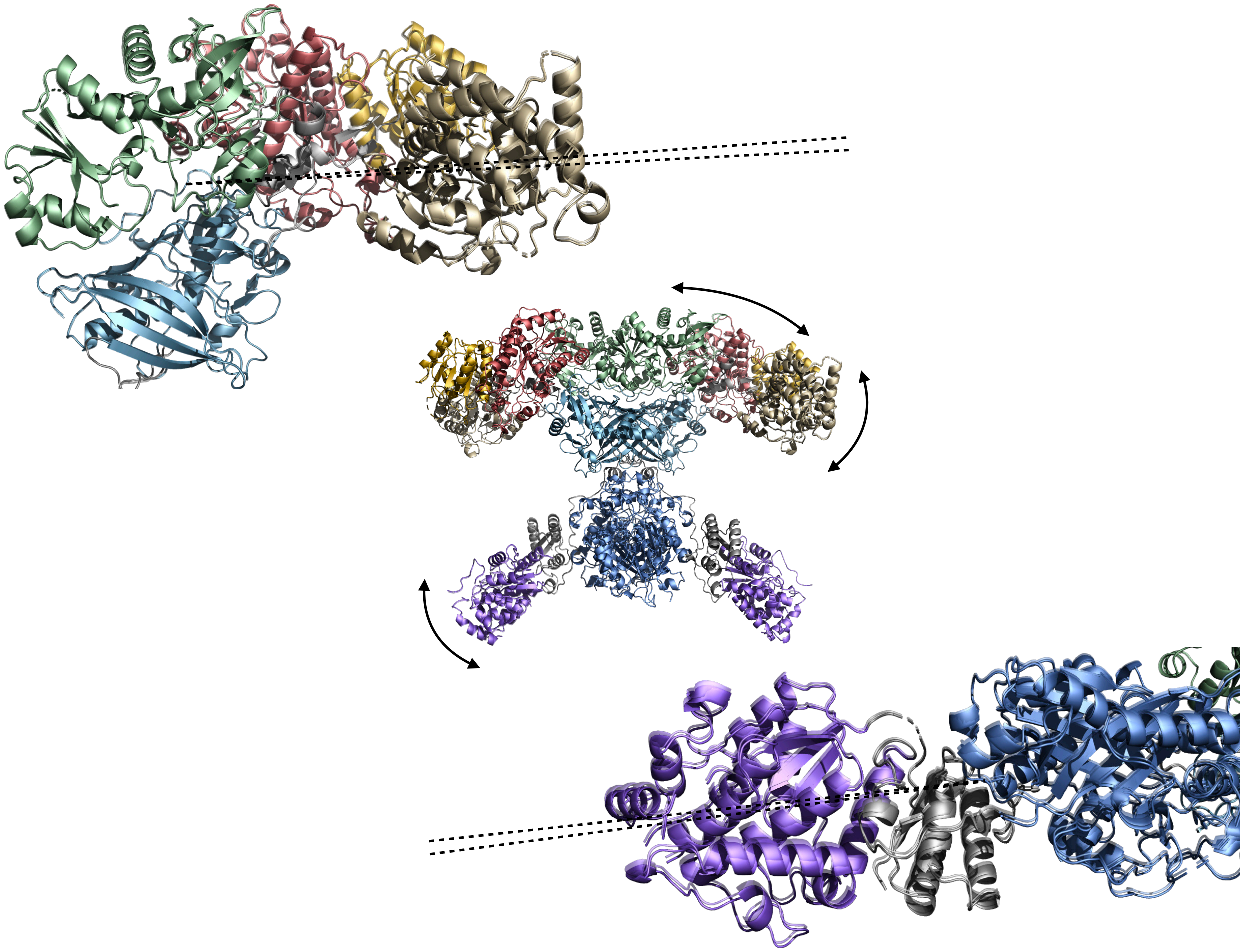

Supplementary Fig. 8 Dynamics of the LovB structure. Two models were built based on the two EM maps shown in Supplementary Fig. 5, and superimposed. A ≈0.4° angle difference for each domain was observed between two models, emphasized by dashed lines located in the zoomed-out view of condensing and tailoring regions, indicating subtle motion of the LovB structure.

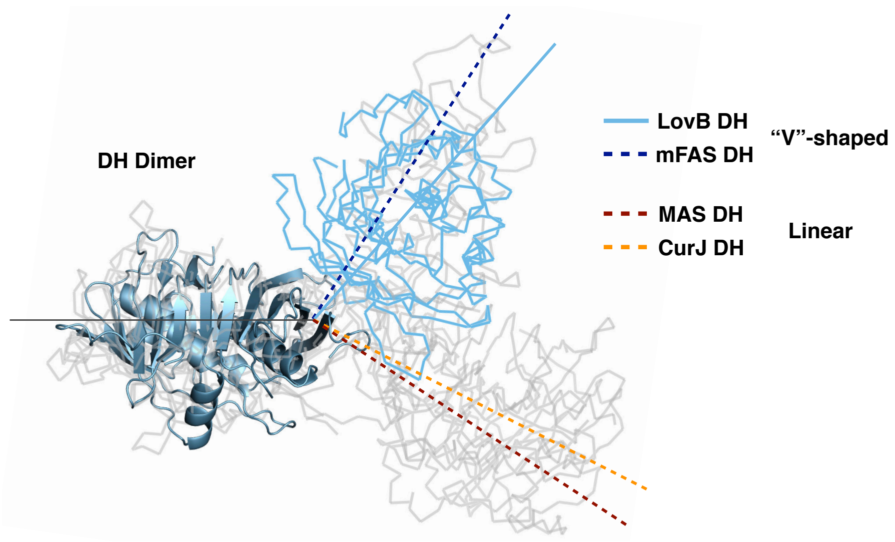

Supplementary Fig. 9 The dimer DH domain organization of LovB compared with its homologs. One domain of the DH dimers from LovB, mFAS, MAS, and CurJ is superimposed. The second domains are shown as ribbons with different colors. The directions of the second domains are indicated with lines.

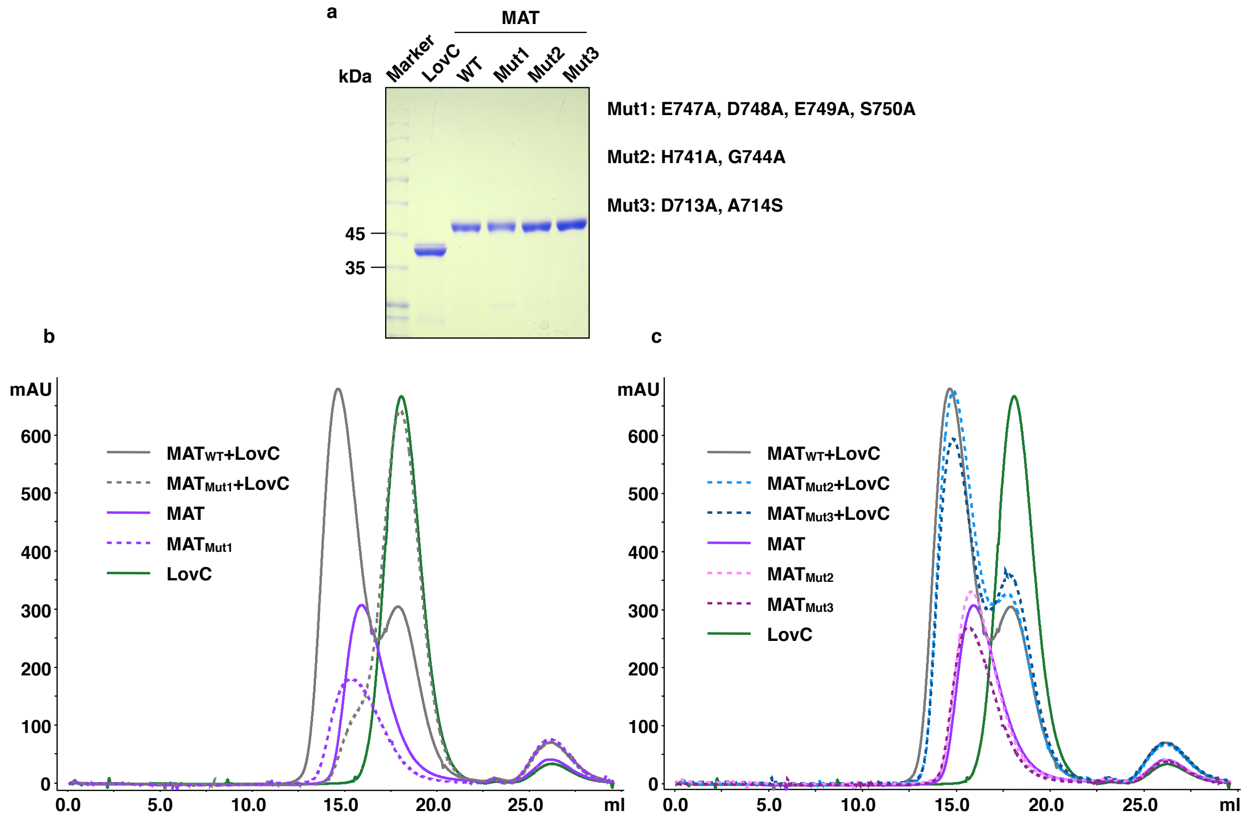

Supplementary Fig. 10 Mutational analysis of the residues in the MAT domain mediating protein-protein interactions. The residues in the MAT domain mediating the interaction with LovC were mutated to alanine. **a** The purity of MAT domain protein mutants was analyzed using SDS-PAGE. One representative result from at least three independent experiments is shown. Source data is provided as a Source Data file. **b** Gel-filtration interaction study of MAT Mut1 with LovC. **c** Gel-filtration interaction study of MAT Mut2-3 with LovC.

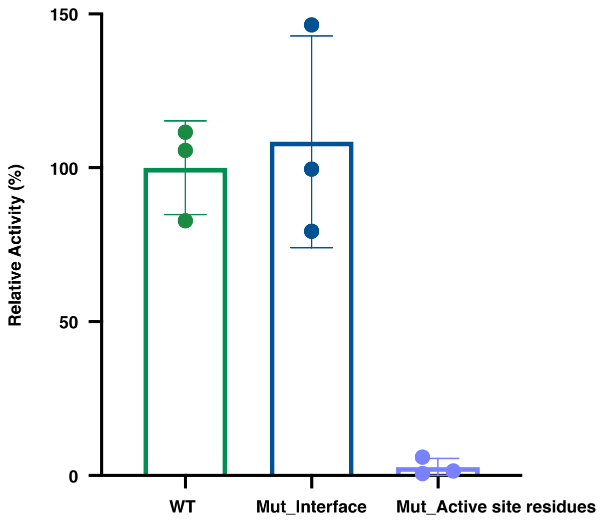

Supplementary Fig. 11 Activity assay of LovC interface mutant. The plot shows relative activities of LovC WT (-•-), the interface mutant (-•-) and the active site residues mutant (-•-). Error bars indicate standard deviations (± SD) from three biologically independent experiments (*n*=3). Source data are provided as a Source Data file.

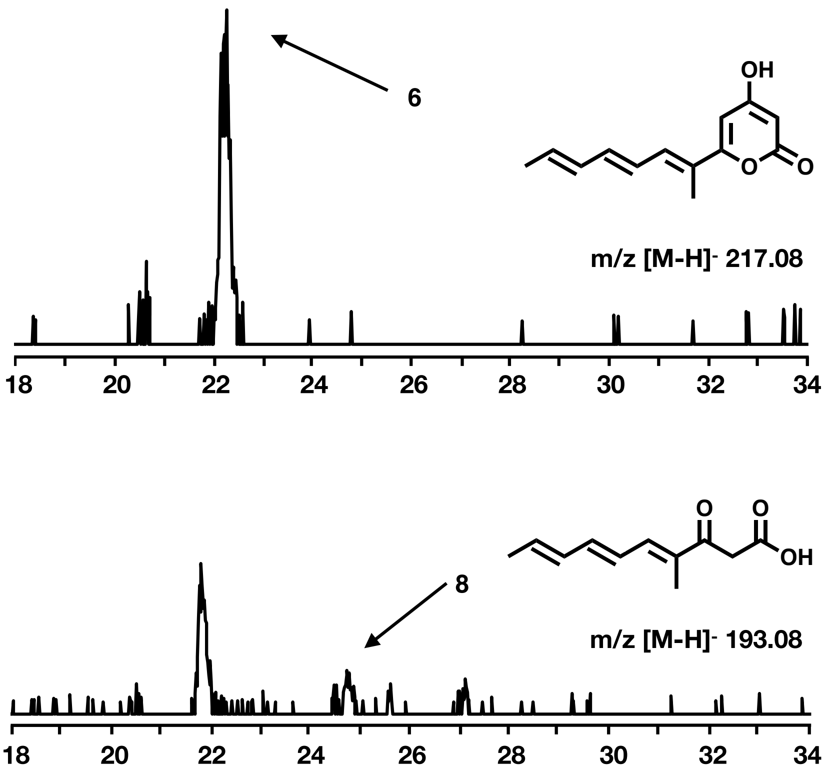

Supplementary Fig. 12 Compounds synthesized by LovB and LovC mutant. HPLC traces showing compounds 6 and 8, which were synthesized by LovB and LovC mutant in the presence of LovG. The compounds were detected using mass spectrometry. These compounds were characterized by Xu. *et al*[1]. Compound 6 has calculated and experimentally determined m/z $[M-H]^-$ values of 217.08 and 217.0873, respectively. Compound 8 has calculated and experimentally determined m/z $[M-H]^-$ values of 193.08 and 193.0873, respectively.

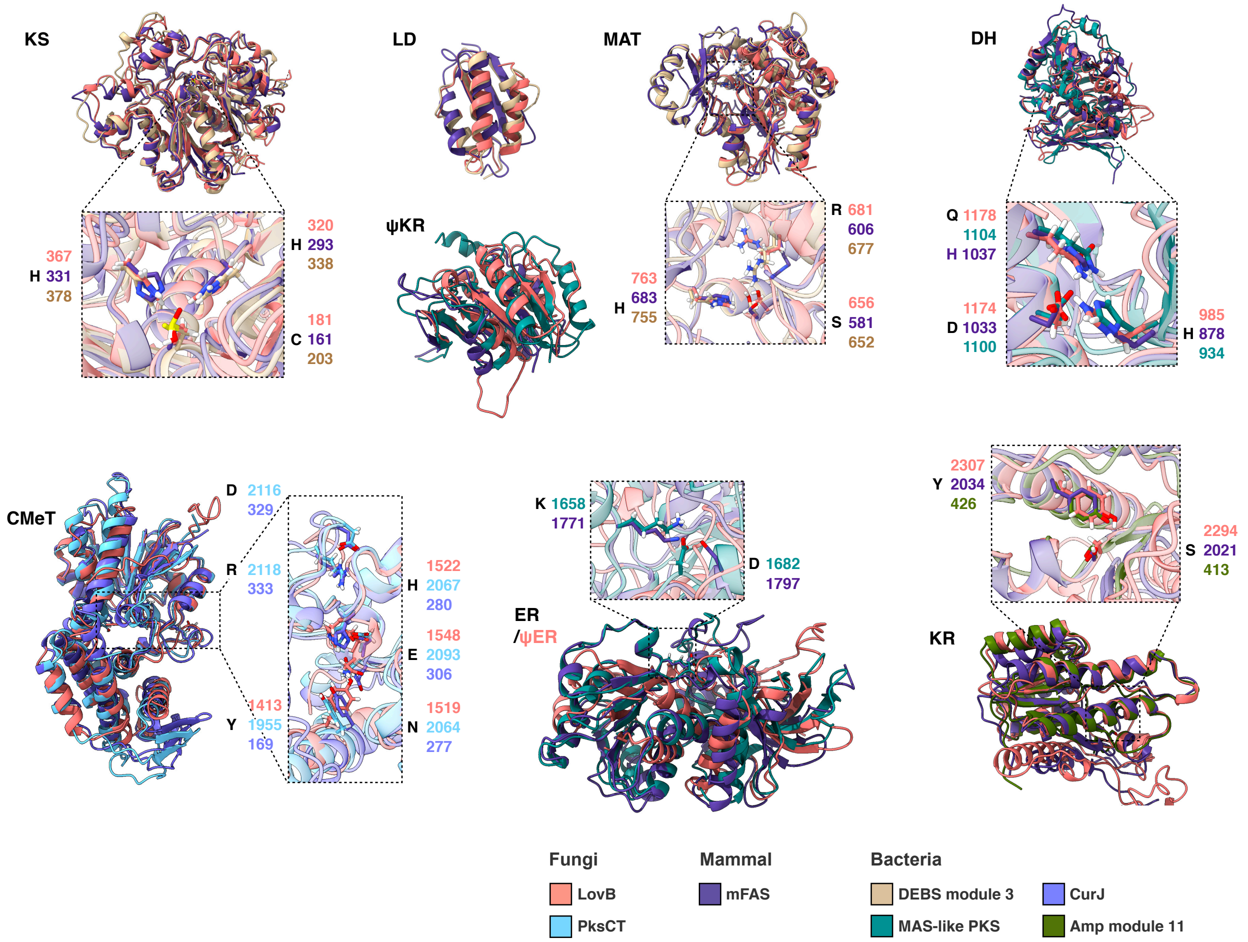

Supplementary Fig. 13 Structural comparison of LovB domains with their homologs. Superposition of the ribbon domain structures of LovB (salmon) with their homologous crystal structures of domains from fungi, mammals or bacteria. Conserved active site residues are labeled (black) with residue numbers colored in accordance with structures. The PDB ID codes of the homologs are 2vz8, 6c9u (KS-LD-MAT); 5bp4, 2vz8 (DH, ψKR, ER); 5mpt, 5thy (CMeT); and 2vz8, 4l4x (KR).

Supplementary Table 1. Strains and Plasmids used in the study.

**Strain**		
	**Description**	**Reference**
S. cerevisiae BJ5464-NpgA	Genotype： MAT α *ura3-52 trp1 leu2-delta1 his3-delta200 pep4::*HIS*3 prb1-delta1.6*R *can1* GAL *A.terreus npg*A	
BL21(DE3)	F^{-} *omp*T *hsd*S(rB^{-} mB^{-}) *dcm*+ Tet^{r} *gal*λ(DE3) *end*A *lon hsd*SB (rB^{-} mB^{-})[*lac*I *lac*UV-T7 *gene*1 *ind*1 *sam*7 *nin*5]	Stratagene
DH10B	F^{-} *end*A1 *rec*A1 *gal*U *gal*K *deo*R *nup*G *rps*L Δ*lac*X74 Φ80*lac*ZΔM15 *ara*D139 Δ(*ara,leu*)7697 *mcr*A Δ(*mrr-hsd*RMS-*mcr*BC) λ^{-}	Invitrogen
**Plasmids**		
pXW_LovBcH	Expression plasmid for C-terminus hexahistidine tagged LovB constructed to a 2μ-based yeast-*E. coli* shuttle plasmid (2μ origin, *E. coli ori*, β-*lac*, *ura*3, ADH2 promoter and terminator, C6His, $Camp^{R}$)	
pET28_LovCcH	Expression plasmid for C-terminus hexahistidine tagged LovC (constructed in pET28a (+) vector with Kan^{R} markers)	

Supplementary Table 2. Mutational plasmids and primers used in the study.

**Mutational plasmids and primers**				
**Plasmid**	**Description**	**Primer**	**Sequence**	
pLovB_MATcH	MAT domain	V28_Mt55_S	GTGGAATCCCACCATCATCACCACCATTA	Vector
		V28_Mt55_A	TGGCTCCATGCCCATGGTATATCTCCTTC	
		Mat28_18_S	ACCATGGGCATGGAGCCAGAGCAAAACC	MAT Fragment
		Mat28_18cH_A	ATGATGGTGGGATTCCACCCAGTAGCGA	
pLovB_MAT$_{mut1}$cH	MAT Mut1 (E747A, D748A, E749A, S750A)	M1-10EDES4A-S	CCGCAGCTGCCACTTTTGCGAGACTGCTCA	
		M1-10EDES4A-A	GCAGCTGCGGCCAACATGCCCTTCAGGTGA	
pLovB_MAT$_{mut2}$cH	MAT Mut2 (H741A, G744A)	M2-10HG2A-S	CTCTGAAGGCCATGTTGGAGGATGAGTCCACTT	
		M2-10HG2A-A	GCCTTCAGAGCATCAATTGCGTTCGCGTCG	
pLovB_MAT$_{mut3}$cH	MAT Mut3 (D713A, A714S)	M3-10DA2AS-S	CTTCCTTTGAGGGCCGCATCTGCGT	
		M3-10DA2AS-A	TCAAAGGAAGCCAACTCGCAGATCTCTTG	
pLovC$_{mut}$cH	LovC Mut (T271L, R272I, K273G, M274A)	TRKM to LIGA-S	GATTGGTGCAGTCACGACCGACTGGACCCTGG	
		TRKM to LIGA-A	CACCAATCAGGGCCGCGTGTTCAGGGAACG	

Supplementary Table 3. Cryo-EM data collection, processing and validation statistics.

	LovB+C Dataset 1+ Dataset 2	
	LovB (EMD-30434) (PDB 7CPX)	LovB with LovC (EMD-30435) (PDB 7CPY)
**Data collection and processing**		
Magnification	22500	22500
Voltage (kV)	300	300
Electron exposure (e-/Å^2)	60.8	60.8
Defocus range (μm)	-1.5 to -2.5	-1.5 to -2.5
Pixel size (Å)	1.00	1.00
Symmetry imposed	C2	C1
Final particle images (no.)	205,047	83,573
Map resolution (Å)	2.91	3.6
FSC threshold	0.143	0.143
Map resolution range (Å)	2.7-7.7	3.3-16.4
Map sharpening B-factor (Å)	-79.8	-74.2
**Model composition**		
Chains	2	4
Non-hydrogen atoms	35304	40788
Protein residues	4524	5238
Ligand	2	2
**R.m.s. deviations**		
Bond lengths (Å)	0.004	0.007
Bond angles (°)	0.979	1.077
**Validation**		
MolProbity score	2.21	2.12
Clashscore	9.39	8.93
Favored rotamers (%)	91.86	92.05
Poor rotamers (%)	2.94	2.91
**Ramachandran plot**		
Favored (%)	94.86	96.04
Allowed (%)	5.05	3.88
Disallowed (%)	0.09	0.08

Supplementary Reference

1. Xu, W. *et al.* LovG: the thioesterase required for dihydromonacolin L release and lovastatin nonaketide synthase turnover in lovastatin biosynthesis. *Angew. Chem. Int. Ed. Engl.* **52,** 6472–6475 (2013).
