# Supplementary material for: Structural basis for the biosynthesis of lovastatin
Source: Nat Commun. 2021 Feb 8;12:867. doi: 10.1038/s41467-021-21174-8 (PMC7870829; doi:10.1038/s41467-021-21174-8)
Supplement: Supplementary file 3 — Description of Additional Supplementary Files [file 41467_2021_21174_MOESM3_ESM.docx]

**Description of Additional Supplementary File**

**File Name**: Supplementary Data 1

**Description**: The LovB-C interface simulated by computational docking analysis using RosettaDock. The detail of interacting residues is shown in Fig. 4c.
